# Associations between self-reported sleep duration and incident cardiovascular diseases in a nationwide prospective cohort study of Chinese middle-aged and older adults

**DOI:** 10.3389/fcvm.2024.1474426

**Published:** 2024-12-09

**Authors:** Qing Zhao, Yuan Zhu, Yu Zhang, Huanyuan Luo, Yantao Ma, Xiaoshan Chen, Jiaming Gu, Lizhi Wang

**Affiliations:** ^1^ School of Health Management, Southern Medical University, Guangzhou, China; ^2^ School of Nursing, Nanjing University of Chinese Medicine, Nanjing, China; ^3^ School of Humanities and Health, Changzhou Vocational Institute of Textile and Garment, Changzhou, China; ^4^ Southern Medical University Institute for Global Health (SIGHT), Dermatology Hospital of Southern Medical University (SMU), Guangzhou, China; ^5^ Shenzhen International Graduate School, Tsinghua University, Shenzhen, China; ^6^ Southern Medical University Center for World Health Organization Studies, Southern Medical University, Guangzhou, China; ^7^ Research Base for Development of Public Health Service System of Guangzhou, Southern Medical University, Guangzhou, China; ^8^ Key Laboratory of Philosophy and Social Sciences of Guangdong Higher Education Institutions for Health Polices Research and Evaluation (2015WSY0010), Guangzhou, China; ^9^ Humanities and Social Sciences Popularization Base for Public Health Emergency and Health Education, Southern Medical University, Guangzhou, China

**Keywords:** sleep duration, cardiovascular disease, age, gender, CHARLS

## Abstract

**Purpose:**

This study explores the correlation between sleep duration and cardiovascular disease (CVD) among middle-aged and older adults in China. Furthermore, we aim to investigate the association between sleep duration and incident CVD in this population, while assessing potential variations across different age and gender subgroups.

**Methods:**

Utilizing data from the nationwide prospective survey of the China Health and Retirement Longitudinal Study (CHARLS) conducted in 2011, 2013, 2015, and 2018, involving 17,596 participants aged 45 years and above, we employed Cox proportional hazards regression models. These models were used to examine the impact of baseline sleep duration on CVD, considering age (middle-aged/elderly) and gender (male/female) groups.

**Results:**

Over the 8-year follow-up, 2,359 CVD events were recorded. Compared to individuals sleeping 6–8 h per day, a short sleep duration (≤6 h/day) was significantly associated with an increased risk of CVD (HR: 1.17, 95% CI: 1.03–1.33). Subgroup analysis revealed a more pronounced relationship in participants aged ≥60 years, where both short sleep duration (≤6 h/day) (HR: 1.17, 95% CI: 1.02–1.35) and long sleep duration (>8 h/day) (HR: 1.20, 95% CI: 1.02–1.41) were significantly associated with an elevated risk of CVD. Specifically, among female participants, short sleep durations (≤6 h/day) was significantly associated with CVD (HR: 1.24, 95% CI: 1.05–1.47).

**Conclusion:**

Short sleep durations can serve as predictive factors for CVD in China's population aged 45 and above, particularly among elderly female participants. Our study underscores the importance of considering sleep health as a critical aspect when formulating strategies for enhancing CVD prevention.

## 1 Introduction

Despite significant advancements in healthcare and our understanding of the potential causes of cardiovascular diseases (CVD), it continues to be a major contributor to global mortality and the loss of disability-adjusted life years (DALYs) (1). Cardiovascular diseases (CVD) represent the most prevalent noncommunicable conditions globally, accounting for approximately one-third of all deaths on a global scale (2). This phenomenon is particularly pronounced in low- and middle-income countries (3, 4). In China, CVD stand as a significant factor contributing to the overall disease burden, accounting for 46.74% and 44.26% of all deaths in rural and urban areas, respectively. Two out of every five deaths are attributed to CVD, impacting an estimated 330 million individuals in China (5). Furthermore, the increasing prevalence of established risk factors such as obesity, diabetes, excessive alcohol consumption, and smoking will only further elevate the risk of CVD (4, 5). Given the high incidence, mortality rates, and substantial disability associated with CVD, it is imperative to identify additional potential risk factors, particularly modifiable lifestyle factors. This will enable the formulation of more effective strategies for CVD prevention.

Sleep, an integral element of our daily existence, assumes a critical role in preserving metabolic equilibrium and overall human well-being. Nevertheless, deviations in sleep duration are increasingly identified as behavioral risk factors linked to diverse health outcomes, including obesity, diabetes, and, notably, all-cause mortality. Recent investigations have sought to unravel the intricate relationship between sleep duration and cardiovascular disease (CVD) risk. Nonetheless, these studies grapple with inherent limitations, primarily stemming from the specificity of study populations to particular diseases, such as diabetes (6), or confined to a single city rather than a more representative sample of the entire population (7). For instance, Ke et al. investigated the association between sleep duration and increased CVD risk in southern China (7). However, the limited sample source and size in their study may raise questions about its representativeness. Secondly, it is crucial to choose the appropriate statistical method for research outcomes. Lin et al. examined the relationship between sleep duration and CVD using data from a nationwide health survey (8). However, this study employed multivariable logistic regression, which has several limitations in handling survival data (9), such as not considering time factors and proportional hazards assumption. Thirdly, some studies have been conducted in Western populations, which may have different lifestyle habits and sleep patterns compared to the Chinese population (10). Therefore, it is crucial to further investigate and confirm the relationship between sleep duration and CVD in a more diverse Chinese population through a nationwide prospective cohort study.

In addition, it is important to recognize that sleep patterns naturally evolve with the aging process, and individuals in middle and older age groups are more susceptible to sleep disturbances compared to younger individuals (11, 12). Moreover, studies have demonstrated gender disparities in the risk of cardiovascular disease and diabetes (13, 14). Importantly, limited research has specifically explored the association between self-reported sleep duration and these cardiovascular conditions, particularly in relation to gender-specific differences. Chen et al. utilized logistic regression models to investigate these associations using the US adults data from the National Health and Nutrition Examination Survey (NHANES). However, as this was a cross-sectional study and did not utilize a Chinese sample, further research is warranted to longitudinally examine how age and gender contribute to these relationships within a more diverse Chinese population. Hence, our hypothesis posits that age and gender could potentially exert influence on the correlation between sleep duration and the incidence of cardiovascular disease (CVD).

In an effort to bridge this knowledge gap, the present study endeavors to leverage data from a nationally representative longitudinal survey to scrutinize the correlation between sleep duration and the incidence of CVD. Furthermore, we have structured the analysis to account for variations based on age and gender. Anticipated as a result of this study are valuable insights intended to inform the judicious regulation of sleep duration among middle-aged and older individuals, thereby contributing to the prevention of CVD.

## 2 Materials and methods

### 2.1 Study population

The China Health and Retirement Longitudinal Study (CHARLS) is a nationwide, community-based, representative health survey focused on middle-aged and elderly adults in China. CHARLS aims to comprehensively assess primary health issues and the socioeconomic determinants associated with China's aging population. In the initial survey, a total of 17,596 participants were recruited during the baseline phase, conducted from 2011 to 2012 (wave 1). Subsequent follow-ups took place in 2013–2014 (wave 2), 2015–2016 (wave 3), and 2017–2018 (wave 4). More extensive information about CHARLS' design and participant profiles can be found elsewhere (15). The Institutional Review Board of Peking University approved CHARLS, and all participants provided written informed consent.

For analytical purposes, we excluded individuals meeting any of the following criteria: (1) those without age information or under the age of 45 (*n* = 218), (2) those without data on nighttime sleep or daytime naps at baseline (*n* = 1,487), (3) those who had already experienced CVD at baseline (*n* = 2,374), (4) we excluded individuals who did not provide information on the interview year and month. (*n* = 798).Ultimately, the analytic sample consisted of 12,719 subjects (Supplementary Figure S1).

### 2.2 Assessment of sleep duration

In CHARLS, data on nighttime sleep was collected through the following question: “During the past month, how many hours of actual sleep did you get at night? (average hours for one night)”. Daytime naps were assessed using the question: “During the past month, how long did you take a nap after lunch every day?” Following the approach of previous studies (16, 17), sleep duration was determined as the total daily sleep duration, which was calculated by combining nighttime sleep and daytime nap duration. Specifically, participants were classified into three categories in this study: short sleep duration (≤6 h/day), average sleep duration (6–8 h/day), and long sleep duration (>8 h/day).

### 2.3 Assessment of incident CVD

The study outcome was incident CVD events. In accordance with previous studies (18, 19), incident CVD events were assessed by the following standardized questions: “Have you been told by a doctor that you have been diagnosed with a heart attack, coronary heart disease, angina, congestive heart failure, or other heart problems?” or “Have you been told by a doctor that you have been diagnosed with a stroke?”. This definition aligns with a previous study conducted within the CHARLS framework. Participants who reported heart disease or stroke during the follow-up period were categorized as having incident CVD. The date of CVD diagnosis was documented to fall between the date of the last interview and the date of the interview reporting an incident CVD.

### 2.4 Covariates

Covariates were assessed at baseline, which included age (continuous, years), sex, residence (urban/rural), education levels (less than lower secondary/upper secondary & vocational training/tertiary), household income per capita (continuous, ¥), self-reported status of smoking and drinking (ever/never), instrumental activities of daily living (IADL) impairments (yes/no), basic activities of daily living (BADL) impairments (yes/no), body mass index (BMI), hypertension (yes/no), as well as the self-reported history of cancer (yes/no), diabetes/high blood sugar (yes/no), chronic lung diseases (yes/no), heart problems (yes/no), dyslipidemia (yes/no), arthritis (yes/no), liver disease (yes/no), kidney disease (yes/no), digestive disease (yes/no), asthma (yes/no), memory-related disease (MRD, yes/no) and psychiatric problems (yes/no). Household income per capita was calculated through total household income in yuan divided by family size. IADL consists of doing housework, managing money, shopping for groceries, taking pills and cooking. BADL consists of dressing, showering, eating, toileting, controlling urination and defecation, as well as getting in and out of bed. Participants with incapable of any of situations mentioned above would be classified as experiencing IADL or BADL impairments.

### 2.5 Statistical analysis

Continuous variables were reported as mean ± standard deviation (SD) or as median (25th–75th percentile), while categorical variables were presented as frequency (proportion). To assess the significance of differences in baseline characteristics across sleep duration groups, the Wilcoxon rank-sum test was employed for continuous variables, and the Pearson chi-square test was used for categorical variables. Kaplan-Meier curves and log-rank tests were utilized to illustrate the cumulative risk of incident CVD among the three sleep duration groups. Multivariate Cox proportional hazards regression models were employed to calculate hazard ratios (HRs) and 95% confidence intervals (CIs) to explore the association between sleep duration and incident CVD. The reference group for sleep duration was individuals with an average sleep duration of 6–8 h per day. The fully adjusted model included the following covariates: age, sex, place of residence, education level, household income per capita, smoking and drinking status, BADL/IADL impairments, BMI, and self-reported history of 12 chronic diseases. Proportional hazards assumptions were assessed. Participants contributed person-years from the date of their baseline interview until the date with recorded information on new-onset CVD, death, loss to follow-up, or the last CHARLS survey, whichever event occurred first.

Restricted cubic spline analysis was conducted to evaluate the dose-response relationship between sleep duration and incident CVD. The optimal number of knots (3–7) was determined by Akaike's information criterion (AIC), with a smaller AIC indicating a better fitting (20). Furthermore, in order to investigate the moderating effects of age and gender on such association, repeated subgroups analyses were further conducted by age (<60 years, middle-aged; ≥60 years, elderly) and gender (male; female). All data analyses were performed with R version 4.3.0 and two-sided *P-*value < 0.05 was deemed significant.

## 3 Results

Among the 12,719 participants included in this analysis, the number (proportion) of individuals with different sleep durations were as follows: 4,801 (37.7%) with ≤6 h/day, 5,034 (39.6%) with 6–8 h/day, and 2,884 (22.7%) with >8 h/day. Participants with either shorter or longer sleep durations were more likely to be older, live in rural areas, have the lowest income and education level. Additionally, individuals with shorter sleep durations tended to be more frequently female, non-smokers, and non-drinkers. The prevalence rates of most chronic diseases exhibited statistically significant differences among the three sleep duration groups, with the exceptions of hypertension, diabetes or high blood sugar, cancer, memory-related disease, psychiatric problems, dyslipidemia and Asthma (all with *P*-values < 0.05; Table 1).

**Table 1:** Characteristics of the study population.

Variables (%)	Sleep duration (h/day)*			*P*-value
	Short (≤6 h)	Average (6–8 h)	Long (>8 h)	
N, *N* (%)	4,801 (37.7)	5,034 (39.6)	2,884 (22.7)	
Age (years), median (Q1-Q3)	65.0 (59.0 to 73.0)	63.0 (56.0 to 70.0)	64.0 (56.0 to 72.0)	<0.001
Male, *N* (%)	2,073 (43.2%)	2,527 (50.2%)	1,492 (51.7%)	<0.001
Rural residency, *N* (%)	3,150 (65.6%)	2,966 (58.9%)	1,843 (63.9%)	<0.001
Education, *N* (%)				
Less than lower secondary education	4,409 (91.8%)	4,294 (85.3%)	2,550 (88.4%)	<0.001
Upper secondary & vocational training	351 (7.3%)	617 (12.3%)	283 (9.8%)	
Tertiary	41 (0.9%)	123 (2.4%)	51 (1.8%)	
Household income per capita (¥), median (Q1-Q3)	3,000.0 (780.0 to 8,200.0)	4,280.0 (1,160.0 to 10,200.0)	3,700.0 (945.4 to 9,570.0)	<0.001
BMI (Q1-Q3)	22.60 (20.41 to 25.17)	23.21 (20.98 to 25.74)	23.19 (20.97 to 25.80)	<0.001
Smoking, *N* (%)	1,771 (36.9%)	2,014 (40.0%)	1,193 (41.4%)	<0.001
Drinking, *N* (%)	1,813 (37.8%)	2,017 (40.1%)	1,184 (41.1%)	<0.001
BADL impairments, *N* (%)	929 (19.5%)	467 (9.4%)	329 (11.6%)	<0.001
IADL impairments, *N* (%)	1,147 (24%)	697 (13.9%)	466 (16.2%)	<0.001
Hypertension, *N* (%)	1,022 (21.4%)	1,023 (20.4%)	639 (22.2%)	0.150
Diabetes/high blood sugar, *N* (%)	218 (4.6%)	232 (4.6%)	145 (5.1%)	0.614
Cancer, *N* (%)	39 (0.8%)	37 (0.7%)	21 (0.7%)	0.879
Lung disease, *N* (%)	467 (9.7%)	365 (7.3%)	240 (8.3%)	<0.001
MRD, *N* (%)	61 (1.3%)	41 (0.8%)	36 (1.3%)	0.058
Psychiatric problems, *N* (%)	59 (1.2%)	42 (0.8%)	23 (0.8%)	0.074
Arthritis, *N* (%)	1,825 (38.1%)	1,364 (27.1%)	764 (26.5%)	<0.001
Dyslipidemia, *N* (%)	339 (7.2%)	415 (8.4%)	215 (7.5%)	0.091
Liver disease, *N* (%)	174 (3.6%)	125 (2.5%)	74 (2.6%)	<0.001
Kidney disease, *N* (%)	251 (5.3%)	220 (4.4%)	107 (3.7%)	<0.006
Digestive disease, *N* (%)	1,185 (24.7%)	907 (18.0%)	500 (17.4%)	<0.001
Asthma, *N* (%)	197 (4.1%)	162 (3.2%)	100 (3.5%)	0.057

MRD, memory-related disease.

* Sleep duration was calculated as nighttime sleep duration plus daytime nap duration.

From 2011 to 2018, a total of 2,359 incident CVD cases were reported. In the entire population, the incidence rates of CVD for the three sleep duration groups were 36.37, 29.45, and 32.4 per 1,000 person-years, respectively. Within the subgroups based on age and gender, individuals with short sleep duration continued to exhibit relatively higher incidence rates of CVD compared to those with average and long sleep durations (Table 2). Furthermore, when compared to individuals with an average sleep duration (6–8 h/day), those with either short or long sleep durations had a significantly higher risk of experiencing a CVD (*P* < 0.0001). Notably, individuals with short sleep duration (≤6 h/day) had the lowest probability of remaining CVD-free compared to those in the other groups (Supplementary Figure S2).

**Table 2:** Incidence rate of CVD per 1,000 person-years by sleep duration.

Incidence	Sleep duration (h/day)^a^		
	≤6	6–8	>8
Total			
Incident case (*n*)	974	852	533
Follow-up (person-years)	26,777	28,932	16,449
Incidence rate of CVD	36.37	29.45	32.40
Age ≥ 45 and < 60 years (*n*)			
Incident case (*n*)	190	220	117
Follow-up (person-years)	7,236	10,540	5,957
Incidence rate of CVD	26.26	20.87	19.64
Age ≥ 60 years (*n*)			
Incident case (*n*)	784	632	416
Follow-up (person-years)	19,541	18,393	10,493
Incidence rate of CVD	40.12	34.36	39.65
Male (*n*)			
Incident case (*n*)	356	398	267
Follow-up (person-years)	11,679	14,439	8,443
Incidence rate of CVD	30.48	27.56	31.62
Female (*n*)			
Incident case (*n*)	618	454	266
Follow-up (person-years)	15,098	14,494	8,006
Incidence rate of CVD	40.93	31.32	33.23

^a^ Sleep duration was calculated as nighttime sleep duration plus daytime nap duration.

Additionally, multivariable-adjusted restricted cubic splines analysis revealed a nonlinear relationship between sleep duration and the occurrence of CVD (*P* < 0.001), as depicted in Supplementary Figure S3. Within the range of sleep durations less than 8.03 h, the hazard ratio gradually decreased, but it started to increase after 8.03 h. This suggests that there is a non-linear relationship between sleep duration and the risk of incident CVD, with the lowest risk observed within this specific range of sleep duration.

The results of the Proportional Hazards Assumption analysis indicated that all independent variables had *p*-values greater than 0.05, suggesting no violation of the proportional hazards assumption. Compared to individuals in the reference group with 6–8 h of sleep per day, those with short sleep duration exhibited a higher crude hazard ratio (HR) of 1.23 (95% CI: 1.12–1.35) for developing incident CVD. After adjusting for a series of confounding factors, the multivariate-adjusted relationship between short sleep duration and incident CVD remained positive, with the adjusted HR being attenuated to 1.17 (95% CI: 1.03–1.33). However, the association between long sleep duration and incident CVD was not significant (*P >* 0.05) (Table 3).

**Table 3:** Associations between sleep duration and risk of CVD.

Incident CVD	Sleep duration (h/day)^a^		
	≤6	6–8	>8
Cases [*n* (%)]	974 (7.7%)	852 (6.7%)	533 (4.2%)
Unadjusted	1.23 (1.12–1.35)	1 (ref)	1.10 (0.98–1.22)
Model 1^b^	1.22 (1.08–1.36)	1 (ref)	1.18 (1.03–1.35)
Model 2^c^	1.17 (1.03–1.32)	1 (ref)	1.15 (0.99–1.33)
Model 3^d^	1.17 (1.03–1.33)	1 (ref)	1.14 (0.99–1.33)

^a^ Sleep duration was calculated as nighttime sleep duration plus daytime nap duration.

^b^ Model 1: adjusted for age, sex, living place, household income per capita and education level.

^c^ Model 2: Model 1 + smoking status, drinking status, BADL/IADL impairments, BMI.

^d^ Model 3: Model 2 + dyslipidemia, diabetes/high blood sugar, heart problems, cancer, chronic lung disease, memory-related disease, kidney disease, liver disease, arthritis, digestive disease, asthma, hypertension and Psychiatric problems.

Furthermore, subgroup analysis results indicated that this relationship was more pronounced in participants aged 60 years and older, with short sleep duration (≤6 h/day) significantly associated with an elevated risk of CVD (HR: 1.17, 95% CI: 1.02–1.35). Long sleep duration (> 8 h/day) was also significantly associated with an increased risk of CVD (HR: 1.20, 95% CI: 1.02–1.41) in this age group. In male participants, longer sleep duration (>8 h/day) was significantly associated with an increased risk of CVD (HR: 1.26, 95% CI: 1.02–1.55). In female participants, short sleep duration was both significantly associated with an increased risk of CVD (HR: 1.24, 95% CI: 1.05–1.47) (Table 4).

**Table 4:** Stratified analyses of age and gender groups in the association of sleep duration with incident CVD.

Subgroups	*N*	Events, *n* (%)	Adjusted HR (95% CI)
Age			
Age < 60			
≤6	1,231	190	1.16 (0.84–1.61)
6–8	1,770	220	1 (ref)
>8	976	117	0.96 (0.66–1.40)
Age ≥ 60			
≤6	3,570	784	1.17 (1.02–1.35)
6–8	3,264	632	1 (ref)
>8	1,908	416	1.20 (1.02–1.41)
Gender			
Male			
≤6	2,073	92	1.07 (0.87–1.30)
6–8	2,527	62	1 (ref)
>8	1,492	34	1.26 (1.02–1.55)
Female			
≤6	2,728	618	1.24 (1.05–1.47)
6–8	2,507	454	1 (ref)
>8	1,392	266	1.04 (0.84–1.28)

## 4 Discussion

In this nationwide prospective cohort study involving middle-aged and elderly individuals, our findings revealed a positive association between short sleep duration at baseline and the risk of future CVD, even after accounting for several potential confounding factors. Notably, this elevated risk was observed primarily in elderly adults and female.

The association between sleep duration and the risk of cardiovascular disease (CVD) has been examined in numerous studies, yet these investigations come with certain limitations. For instance, a prospective cohort study comprising 8,245 Chinese community residents revealed an independent association between short sleep duration and an increased incidence of CVD (7). However, it is worth noting that the study defines the independent variable based on the past 24 h of sleep time. Extracting the independent variable in this way may lead to overlooking nap times, resulting in an underestimation of total sleep duration (21). In our study, we sum the nap time and nighttime sleep time to calculate total sleep duration more accurately. For instance, individuals with obstructive sleep apnea (OSA) are more likely to have excessive daytime naps along with shorter nighttime sleep (22). Consequently, assessing the association between nighttime sleep and incident cardiovascular disease (CVD) solely among individuals with health events might introduce potential bias. Additionally, it's crucial to note that this study was exclusively conducted in Shanghai, the most developed city in China. This sampling approach has the potential to introduce bias to the research findings. Our contribution lies in being a large-scale population-based study that facilitates the adjustment for a myriad of underlying confounders. This aspect enhances the reliability and scientific validity of our findings in comparison to the limited body of published literature. In contrast, Lin et al (8). explored the association between sleep duration and cardiovascular disease (CVD) by analyzing data from a nationwide health survey. However, the study utilized multivariable logistic regression, a method that comes with several limitations when dealing with survival data. These limitations include the neglect of time factors and the assumption of proportional hazards.

Moreover, aligning with several previous studies (6–8, 10), our extensive nationwide cohort study has substantiated the presence of an association between sleep deprivation and the onset of cardiovascular disease (CVD) in middle-aged and elderly adults residing in the community. Notably, in subgroup analyses by age and gender, it is crucial to emphasize that the positive association between short sleep duration and incident CVD was discerned among elderly adults, contrasting with the absence of such an association among participants aged 45–60 years. This age-specific effect may be correlated with the ongoing demographic transition in China, marked by increasing life expectancy and a low fertility rate (23). Unfortunately, this significant demographic shift has contributed to a rise in the prevalence and mortality of diseases that predominantly affect the elderly, such as cancer (24). Furthermore, it has been documented that sleep issues become more prevalent, and sleep duration decreases with age. Individuals aged 60 and above are more likely to experience reductions in their sleep duration, frequent awakenings, and a decrease in slow-wave sleep. Consequently, elderly participants with shorter sleep durations are at a heightened risk of experiencing CVD in the future.

To encapsulate, the insufficiency of sleep has emerged as a noteworthy public health concern within our modern “24/7” society. Amidst this fast-paced milieu, the coexistence of sleep disorders and the prevalence of multiple chronic conditions unavoidably curtail the amount of sleep accessible to middle-aged and elderly individuals (25–27). The current study emphasizes the significance of conducting a comprehensive evaluation of sleep patterns and habits within the general population to identify individuals at an elevated risk of CVD. Unlike biomarkers, which are often challenging to reverse and intervene, sleep is increasingly acknowledged as a modifiable factor influencing CVD risk. Hence, the implementation of more targeted intervention measures and the provision of improved healthcare services aimed at enhancing the duration and quality of sleep may contribute to reducing the incidence of future cardiovascular diseases. This holds particular relevance for female patients aged 60 and above.

The increased risk of cardiovascular disease (CVD) among individuals with short sleep duration is supported by various underlying pathophysiological mechanisms. Evidence indicates that sleep deprivation may induce inflammation, while adequate sleep may facilitate the return of immune cytokines to baseline levels (28). Sleep can profoundly modify the cardiovascular regulation, and a bidirectional link exists in the interconnection between cardiovascular system and sleep (29). Furthermore, some evidence suggests that both excessive and insufficient sleep durations may disrupt normal circadian rhythms, leading to alterations in sympathetic nervous system activity (30). Elevated sympathetic activity has been associated with symptoms of depression and anxiety and may contribute to the development of atherosclerosis (31, 32), which is strongly linked to CVD. Another potential explanation for this association is that sleep deprivation can affect cortisol levels and lead to the up-regulation of the hypothalamic-pituitary-adrenal (HPA) axis, which in turn may contribute to CVD events (33–35). Additionally, it's worth noting that long sleep duration has been associated with a higher atherosclerotic burden specifically in women (36). However, our subgroup analysis results indicate that women with shorter sleep durations are more likely to be at risk for cardiovascular disease (CVD). Variations in study populations and statistical methods can lead to differing conclusions across research. Nevertheless, even in the absence of statistical significance, there remains a trend suggesting that women with longer sleep durations may also be at risk for CVD. Consequently, gender and sleep duration may have a synergistic effect on the risk of incident CVD through exacerbating common pathways, including the processes of atherosclerosis and vascular stiffening. However, the precise mechanisms underlying the relationship between male and incident CVD, especially in terms of age-related disparities, warrant further investigation.

Notably, the cubic spline model shows the standard U-shaped relationship between sleep duration and CVD risk. However, in the hazard models presented, controlling for disease removed the relationship between sleep duration and incident CVD for long sleep durations, suggesting that short sleep is an independent risk factor, while long sleep may merely be a marker of the presence of disease that increases CVD risk. After controlling for confounding factors, long sleep was found to be unrelated to CVD risk, a conclusion supported by previous studies (37). However, after conducting subgroup analyses based on age and gender, we found that older adults and men who sleep longer are more likely to have CVD risk. This indicates that when assessing CVD risk, it is essential to consider individual characteristics and potential confounding factors in greater detail to more accurately understand the complex relationship between sleep duration and cardiovascular health.

The current study has several acknowledged limitations that warrant consideration. Firstly, both physician-diagnosed incident cardiovascular disease (CVD) and sleep duration relied on self-reported measures. Secondly, after conducting a thorough examination, we found that the CHARLS survey did not include interviews on sleep disorders, specifically obstructive sleep apnea (OSA). Consequently, due to this data limitation, we were unable to assess the confounding impact of OSA on these associations. Consequently, future epidemiological surveys should contemplate collecting more comprehensive information on sleep to facilitate a more thorough investigation of this issue.

In summary, our study elucidates that short sleep duration is linked to a moderate increase in the risk of incident CVD among middle-aged and elderly individuals in China. Moreover, our findings suggest that the age and gender of individuals may modify this relationship. These results underscore the importance of vigilant monitoring of sleep patterns, especially within the context of an aging population, and offer new insights for the development of strategies aimed at mitigating the heightened risk of CVD. The findings of the current study may serve as a reference for siesta and sleep duration in individuals aged ≥ 45 years, advocating for a more rational approach to sleep planning. Future research is imperative to unravel the precise mechanisms underlying the age- and gender-specific aspects of the association between sleep duration and incident CVD.

## Data Availability

Publicly available datasets were analyzed in this study. This data can be found here: https://charls.charlsdata.com/pages/Data/harmonized_charls/zh-cn.html. This analysis uses data or information from the Harmonized CHARLS dataset and Codebook, VERSION D developed by the Gateway to Global Aging Data.
