# Supplementary material for: Associations between self-reported sleep duration and incident cardiovascular diseases in a nationwide prospective cohort study of Chinese middle-aged and older adults
Source: Front Cardiovasc Med. 2024 Dec 9;11:1474426. doi: 10.3389/fcvm.2024.1474426 (PMC11664438; doi:10.3389/fcvm.2024.1474426)
Supplement: Supplementary file 1 [file Datasheet1.pdf]

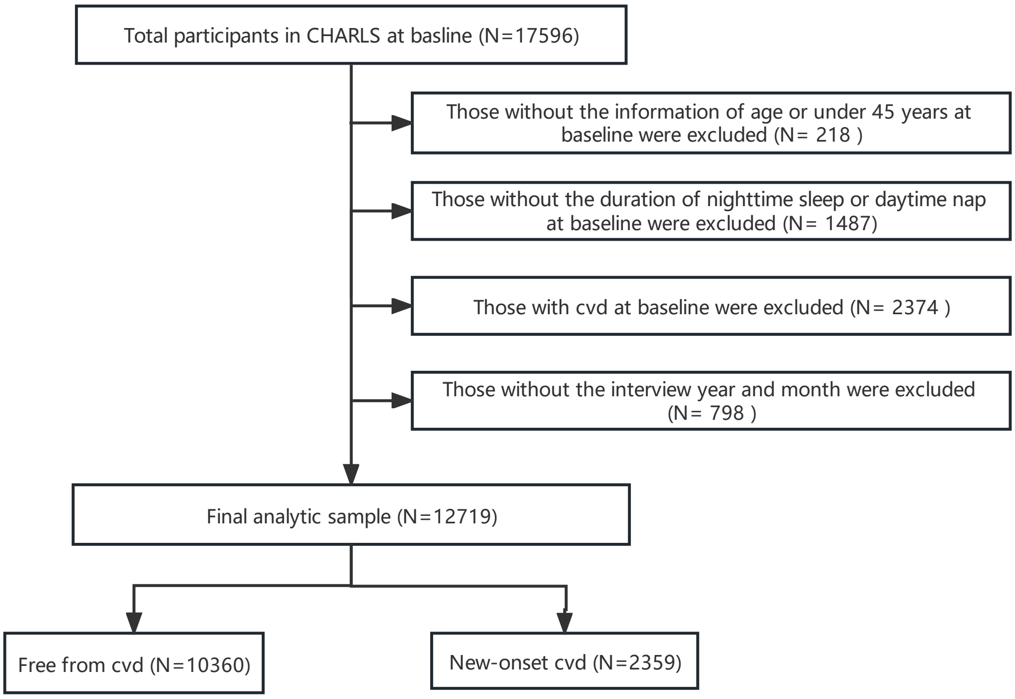

Supplementary Figure 1 Flowchart of subject selection.

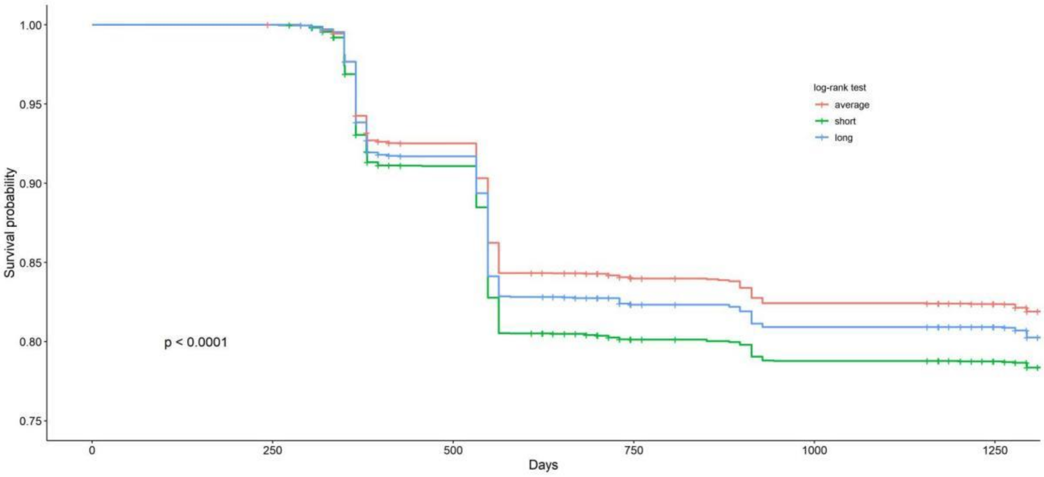

Supplementary Figure 2 Kaplan-Meier curves for the cumulative risk of

CVD by sleep duration categories

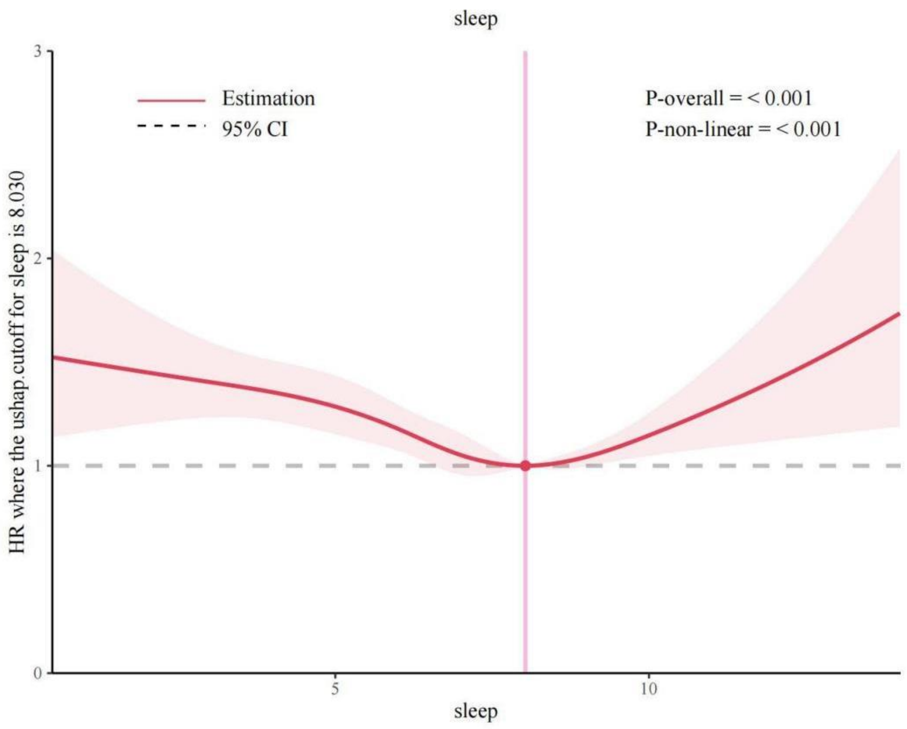

Supplementary Figure 3 Restricted cubic spline analysis of the relationship between sleep duration and incident CVD.
